# Management of acute respiratory failure in interstitial lung diseases: overview and clinical insights

**DOI:** 10.1186/s12890-018-0643-3

**Published:** 2018-05-15

**Authors:** Paola Faverio, Federica De Giacomi, Luca Sardella, Giuseppe Fiorentino, Mauro Carone, Francesco Salerno, Jousel Ora, Paola Rogliani, Giulia Pellegrino, Giuseppe Francesco Sferrazza Papa, Francesco Bini, Bruno Dino Bodini, Grazia Messinesi, Alberto Pesci, Antonio Esquinas

**Affiliations:** 1 Dipartimento Cardio-Toraco-Vascolare, University of Milan Bicocca, Respiratory Unit, San Gerardo Hospital, ASST di Monza, Via Pergolesi 33, 20900 Monza, Italy; 2 UOC di Fisiopatologia e Riabilitazione Respiratoria, AO Ospedali dei Colli Monaldi, Naples, Italy; 3 UOC Pulmonology and Pulmonary Rehabilitation, Istituti Clinici Scientifici Maugeri, IRCCS di Cassano Murge (BA), Cassano delle Murge, Italy; 4 Division of Respiratory Medicine, University Hospital Tor Vergata, Rome, Italy; 5 Dipartimento di Scienze Neuroriabilitative, Casa di Cura del Policlinico, Milan, Italy; 6 Department of Internal Medicine, UOC Pulmonology, Ospedale ASST-Rhodense, Garbagnate Milanese, Italy; 7 Pulmonology Unit, Ospedale Maggiore della Carità, University of Piemonte Orientale, Novara, Italy; 8 Internsive Care Unit, Hospital Morales Meseguer, Múrcia, Spain

**Keywords:** Interstitial lung diseases, Idiopathic pulmonary fibrosis, Acute respiratory failure, Invasive ventilation, Non-invasive ventilation, High-flow nasal cannula

## Abstract

**Background:**

Interstitial lung diseases (ILDs) are a heterogeneous group of diseases characterized by widespread fibrotic and inflammatory abnormalities of the lung. Respiratory failure is a common complication in advanced stages or following acute worsening of the underlying disease. Aim of this review is to evaluate the current evidence in determining the best management of acute respiratory failure (ARF) in ILDs.

**Methods:**

A literature search was performed in the Medline/PubMed and EMBASE databases to identify studies that investigated the management of ARF in ILDs (the last search was conducted on November 2017).

**Results:**

In managing ARF, it is important to establish an adequate diagnostic and therapeutic management depending on whether the patient has an underlying known chronic ILD or ARF is presenting in an unknown or de novo ILD. In the first case both primary causes, such as acute exacerbations of the disease, and secondary causes, including concomitant pulmonary infections, fluid overload and pulmonary embolism need to be investigated. In the second case, a diagnostic work-up that includes investigations in regards to ILD etiology, such as autoimmune screening and bronchoalveolar lavage, should be performed, and possible concomitant causes of ARF have to be ruled out.

Oxygen supplementation and ventilatory support need to be titrated according to the severity of ARF and patients’ therapeutic options. High-Flow Nasal oxygen might potentially be an alternative to conventional oxygen therapy in patients requiring both high flows and high oxygen concentrations to correct hypoxemia and control dyspnea, however the evidence is still scarce. Neither Non-Invasive Ventilation (NIV) nor Invasive Mechanical Ventilation (IMV) seem to change the poor outcomes associated to advanced stages of ILDs. However, in selected patients, such as those with less severe ARF, a NIV trial might help in the early recognition of NIV-responder patients, who may present a better short-term prognosis. More invasive techniques, including IMV and Extracorporeal Membrane Oxygenation, should be limited to patients listed for lung transplant or with reversible causes of ARF.

**Conclusions:**

Despite the overall poor prognosis of ARF in ILDs, a personalized approach may positively influence patients’ management, possibly leading to improved outcomes. However, further studies are warranted.

## Background

**Fig. 1:**
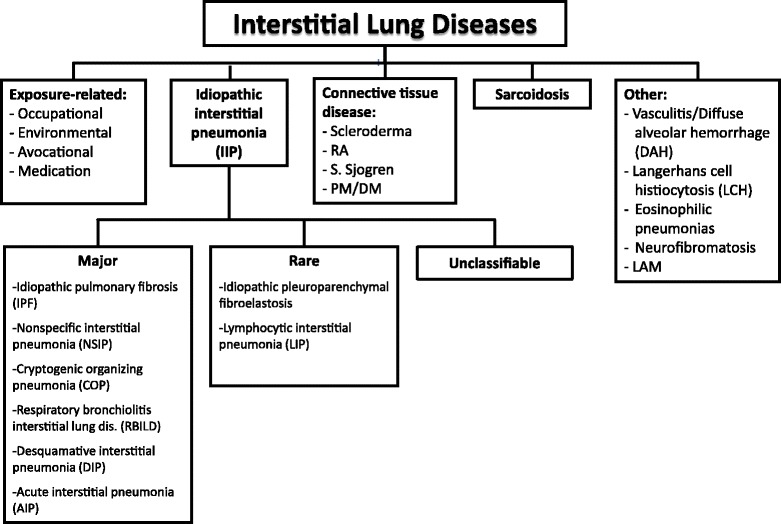
Classification of Interstitial Lung Diseases. Footnotes: RA = Rheumatoid Arthritis; PM/DM = polymyositis/dermatomyositis; LAM = lymphangioleiomyomatosis

Interstitial lung diseases (ILDs) are a heterogeneous group of diseases that includes more than 200 entities characterized by widespread fibrotic and/or inflammatory abnormalities of the lung parenchyma, Fig. 1 [1, 2]. Respiratory failure is a common complication in advanced stages or following acute worsening of ILDs and can be classified on the basis of different parameters, including time of onset (acute or chronic), severity (mild to severe), and causes (reversible or irreversible).

Aim of this review is to evaluate the current evidence in determining the best management of acute respiratory failure (ARF) in ILDs.

## Methods

A search of relevant medical literature in the English language was conducted in Medline/PubMed and EMBASE databases including observational and interventional studies from 1990 through November 2017. Keywords used to perform the research are reported in Table 1. Studies targeting children and editorials, narrative, and conference abstracts have been excluded. For the purpose of this review, any kind of ILD was included in the search.

**Table 1:** Keywords used to perform the research

interstitial lung diseases outcomes, interstitial lung diseases prognosis, interstitial lung diseases (OR IPF OR NSIP OR CTD-ILD OR chronic HP OR acute idiopathic interstitial pneumonia) AND (ventilation OR invasive ventilation OR mechanical ventilation OR invasive mechanical ventilation), non-invasive ventilation AND interstitial lung diseases (OR IPF OR NSIP OR CTD-ILD OR chronic HP OR acute idiopathic interstitial pneumonia), (high flow oxygen OR high-flow nasal cannula OR oxygen therapy OR oxygen supplementation) AND interstitial lung diseases, ((non-invasive ventilation) AND respiratory failure) AND interstitial lung diseases, ((non-invasive ventilation) AND respiratory failure) AND idiopathic pulmonary fibrosis, acute respiratory failure AND interstitial lung disease (OR IPF OR NSIP OR CTD-ILD OR chronic HP OR acute idiopathic interstitial pneumonia), acute respiratory worsening AND interstitial lung disease (OR IPF OR NSIP OR CTD-ILD OR chronic HP OR acute idiopathic interstitial pneumonia), acute exacerbation AND interstitial lung disease (OR IPF OR NSIP OR CTD-ILD OR chronic HP OR acute idiopathic interstitial pneumonia).	

## Results and discussion

### Epidemiology and risk factors

In recent times, ILDs definitions and classifications have been extensively revised [2], therefore it is difficult to provide precise epidemiological data for each class of ILD. In the present review we will mostly address idiopathic pulmonary fibrosis (IPF), which is the most widely studied ILD.

From a study conducted in New Mexico, USA, it was estimated that the prevalence of ILDs was approximately 81 / 100,000 in men and 64 / 100,000 in females [3]. The same study showed a higher incidence in males (31.5 per 100,000 / year) than females (26.1 per 100,000 / year) [3]. Both prevalence and incidence vary greatly depending on the specific type of ILD considered. In particular, although classified as rare diseases, the two most frequently diagnosed ILDs are IPF and sarcoidosis.

IPF has a prevalence of 0.5–27 / 100,000 and an incidence of 0.22–8.8 / 100,000 inhabitants worldwide [4]. When focusing on European data, the prevalence of IPF is 1.25–23.4 / 100,000, and the annual incidence 0.22–7.4 / 100,000 [5]. The incidence of acute exacerbation (AE) of IPF ranges between 0 and 21% according to different cohorts [6–26], and predominantly occurs in advanced stages of the disease [13, 27–29]. This highly variable incidence is probably due to differences in study design (prospective vs retrospective), definition of AE and statistical methodology [30]. Recognized risk factors for AE of IPF (AE-IPF) are lower forced vital capacity and diffusion capacity of carbon monoxide as well as reduced walked distance at 6-min walking test [13, 24, 27, 30–32], worsening respiratory gas exchange [31], higher dyspnea scores [30], greater disease extent on high-resolution computed tomography (HRCT) as well as presence and extent of honeycombing and traction bronchiectasis [33], presence of gastroesophageal reflux disease [34, 35], exposure to air pollution [36], presence of pulmonary hypertension [30], specific genetic variants [37], drug toxicity, bronchoalveolar lavage [38], surgical lung biopsy [39, 40], and surgery, radiotherapy or chemotherapy for concomitant lung cancer [28, 41–43]. Other non-validated risk factors are elevated baseline serum Krebs von der Lungen-6 (KL-6) [44, 45], increased body mass index [24], younger age [30], and concomitant coronary artery disease [27], whereas data on smoke exposure and emphysema as risk factors are discordant [13].

### Outcome of different interstitial lung diseases

IPF is the ILD with the worst prognosis, having a median survival of 2 to 3 years from the time of diagnosis [46]. In 2003 in the USA, the mortality rate related to IPF was 61.2 deaths per 1,000,000 in men and 54.5 deaths per 1,000,000 in women [47]. AE-IPF is the most common cause of mortality in IPF cohorts [48, 49], accounting for over half of all hospitalizations [48] and up to 40% of all deaths [13]. Prognosis of AE-IPF is extremely poor with a in-hospital mortality rate around 50% in less severe patients and higher than 90% in those requiring Intensive Care Unit (ICU) admission [12, 13, 50–55].

Connective tissue disease related ILD (CTD-ILD) has a better prognosis compared to IPF, as emerged from a UK study by *Navaratnam* et al. [56], with a median survival of 6.5 vs 3.1 years in patients with IPF. Among ILDs, sarcoidosis is the one with the best prognosis; *Thomeer* et al. compared the mortality of different forms of ILDs in a tertiary care hospital setting: the 5-year survival of patients with sarcoidosis was 91.6% compared to 69.7% for CTD-ILD and 35% for IPF [57].

Despite the high mortality associated to ARF in all ILDs, IPF showed higher one-year mortality after hospitalization for acute respiratory worsening compared to patients with other fibrotic ILDs, (87% vs 71%, respectively) [58]. Median survival after an AE-IPF ranges between 22 days and 4.2 months [13, 24].

### Underlying pathophysiology of acute respiratory failure in interstitial lung diseases

ARF may occur as an acute/subacute presentation of ILD or may complicate the clinical course of a previously diagnosed ILD or unknown ILD as the result of the rapid decline of respiratory function caused by an accelerated worsening of the underlying interstitial process, the so-called AE, or because of superimposed complications, such as pulmonary thromboembolism, heart failure and infection [13].

AE-ILD might be the consequence of an intrinsic acceleration of the fibroproliferative process or a response to occult or known external events (e.g. infection, micro-aspiration or mechanical stretch such as lung biopsies) [30].

The histopathological hallmark of AE-ILDs, particularly AE-IPF, is diffuse alveolar damage (DAD), superimposed on the histological pattern of the underlying ILD [11, 59–61]. DAD presents histologically with two subsequent phases, an acute/exudative phase followed by an organizing/prolipherative phase, that sometimes evolves in a final fibrotic stage.

The acute exudative phase is characterized by relatively sparse inflammatory cells and predominant hyaline membranes along alveolar septa with accentuation in alveolar ducts. In the organizing/proliferative phase, the hyaline membranes are incorporated into the alveolar septa through phagocytosis by macrophages or granulation tissue formation by proliferating myofibroblasts. Finally, the interstitium is thickened by loose myxoid fibroblastic tissue, causing altered pulmonary gas exchange mainly by diffusion impairment and ventilation-perfusion (V/Q) mismatch.

### Acute respiratory failure etiologies and diagnostic work-up

In patients with ARF and ILD three possible scenarios have to be distinguished:

- ARF in known chronic ILDs

AE was firstly described in IPF patients with reported median survival time after the event of 3 to 4 months [13, 27], even shorter in patients requiring invasive mechanical ventilation (IMV) [62].

The definition of AE-IPF has recently been revised as an acute (less than 1 month in duration) and clinically significant respiratory deterioration in a previously diagnosed IPF patient, associated with the presence of new widespread alveolar infiltrates on HRCT and exclusion of alternative etiologies (including infection, heart failure, pulmonary embolism, and, less frequently, pneumothorax, drug toxicity and diffuse alveolar hemorrhage - DAH -), [30]. Questionably, this new definition removes the distinction between idiopathic and so-called triggered AE because considered irrelevant to patients’ outcome. Nevertheless, identifying the trigger of AE may influence patients’ management [58, 63].

AE may complicate the clinical course of other fibrosing ILDs, such as chronic hypersensitivity pneumonitis (CHP) [64–66], and non-specific interstitial pneumonia (NSIP) both idiopathic and secondary to CTD-ILD [61, 67–69]. The incidence of AE in CTD-ILDs depends on the underlying CTD (as an example, AE are more common in ILD secondary to Rheumatoid Arthritis) and AE may occur regardless of flares of the extrathoracic manifestations and in spite of the immunosuppressive treatment.

**Fig. 2:**
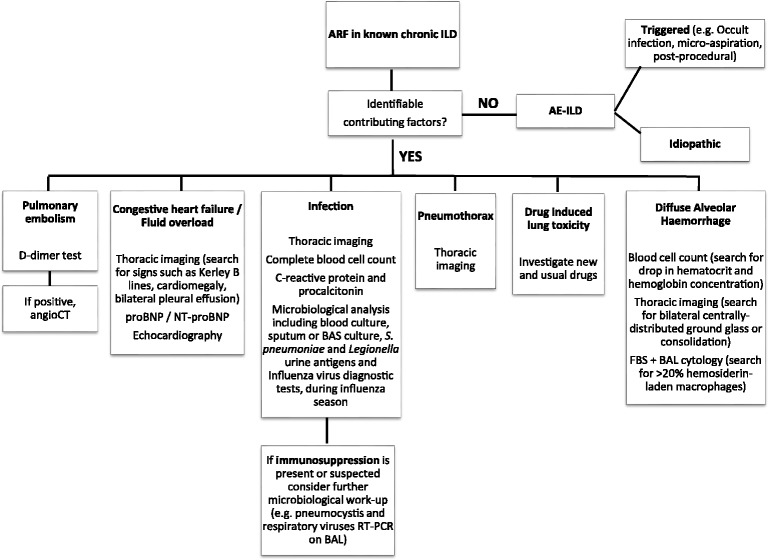
Diagnostic work-up of acute respiratory failure in a known Interstitial Lung Disease. Footnotes: ARF = acute respiratory failure; ILD = interstitial lung disease; AE-ILD = acute exacerbation of ILD; CT = computed tomography; NT-proBNP = N-terminal pro b-type natriuretic peptide; FBS = fiberoptic bronchoscopy; BAS = bronchial aspirate; BAL = bronchoalveolar lavage; RT-PCR = real-time polymerase chain reaction

In clinical practice, a diagnostic work-up based on laboratory exams, CT scan (angioCT if pulmonary embolism is suspected), and bronchoscopy may be recommended in patients with ARF in a known ILD to evaluate all possible scenarios, Fig. 2.

b) Unknown chronic ILD presenting with ARF

Although less commonly than in a previously diagnosed ILD, ARF may also represent the clinical onset of an undiagnosed and unsuspected ILD. Possible ILDs presenting with this clinical manifestation are IPF, NSIP, CHP, CTD-ILDs, DAH and drug-toxicity [11, 13, 68, 70, 71].

In a previously apparently healthy patient presenting with ARF, the assessment of past medical history and symptoms, perhaps underestimated by the patient himself, is mandatory. Physical examination and laboratories (e.g. digital clubbing and polyglobulia) may reveal a long-standing respiratory failure or extrapulmonary manifestations of an underlying CTD or systemic vasculitis. However, the absence of extrapulmonary signs does not exclude an underlying CTD, as pulmonary manifestations may precede by months or years the more typical systemic manifestations, especially in rheumatoid arthritis, systemic lupus erythematosus, and polymyositis-dermatomyositis (lung-dominant ILD) [72]. Therefore, complete autoimmune serology screening, inclusive of myositis-specific autoantibodies, might be helpful.

HRCT, performed as part of the initial assessment of ARF, should be carefully assessed to identify the presence of signs of architectural distortion such as traction bronchiectasis, lung volume loss and honeycombing, considered suggestive of a pre-existing ILD.

Bronchoscopy with bronchoalveolar lavage (BAL) and, in selected cases, lung biopsies may be useful in establishing the diagnosis of the underlying ILD. The indication for bronchoscopic assessment and the choice of the procedure to perform should be carefully discussed in each case, considering ARF severity, the potentially related complications and the risk to trigger an AE.

**Fig. 3:**
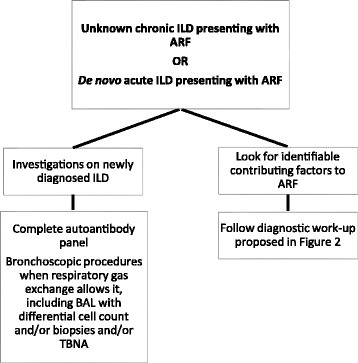
Diagnostic work-up in de novo acute ILD and unknown ILD presenting with ARF**.** Footnotes: ILD = interstitial lung disease; ARF = acute respiratory failure; BAL = bronchoalveolar lavage; TBNA = trans-bronchial needle aspiration

In unknown ILDs presenting with ARF and in de novo acute ILDs, as shown in the next section, the diagnostic work-up should always include both investigations on primary ILD (autoantibody panel and bronchoscopic procedures when respiratory gas exchange allows it) and investigations on concomitant conditions that may cause ARF, as summarised in Fig. 3.

c) De novo acute ILD presenting with ARF

ARF may also represent the clinical onset of a de novo rapidly progressive ILD. In the differential diagnosis the following should be considered: acute interstitial pneumonia (AIP), cryptogenic organizing pneumonia (COP), acute eosinophilic pneumonia (AEP), and drug-induced ILD [73], and, less frequently, acute hypersensitivity pneumonia, DAH in the setting of a de novo vasculitis, de novo CTD-ILD and lepidic adenocarcinoma with lymphangitic carcinomatosis.AIP, formerly known as Hamman-Rich syndrome, is a rare and fulminant form of diffuse lung injury with a clinical presentation similar to acute respiratory distress syndrome (ARDS), whose etiology remains uncertain [74]. The in-hospital mortality rate is greater than 50% and the majority of patients who survive dies within six months of presentation [75, 76].

In 10–15% of cases, COP may present with a rapidly progressive course, mimicking ARDS [77]. The diagnosis is made by ruling out infectious causes of pneumonia and documenting typical pathological changes in tissue obtained by lung biopsies demonstrating fibroblastic polyps, sometimes associated with endoalveolar fibrin deposits, the so-called acute fibrinous organizing pneumonia (AFOP). The majority of patients recovers completely with corticosteroid therapy but relapses are common [78, 79].

AEP occurs in previously healthy individuals and is characterized by pulmonary eosinophilia (more than 25% eosinophils on BAL differential cell count) or eosinophilic pneumonia on lung biopsy with or without peripheral eosinophilia [80].

Many chemotherapeutic (e.g. bleomycin and busulfan) and non-chemotherapeutic agents (e.g. amiodarone, nitrofurantoin, and statins) may cause lung toxicity manifesting with ARF. The diagnosis of a drug-induced acute ILD is challenging, and it is supported by the temporal relationship between the first use of the medication and symptoms onset, spontaneous clinical improvement after the discontinuation of the drug and recurrence of symptoms on re-challenge [81].

Although acute hypersensitivity pneumonitis is most commonly self-limited, it can also present with ARF. It should be suspected when there is a temporal association with the exposure to an inhaled antigen known to trigger hypersensitivity reactions, and in the presence of an upper-lobe predominant process accompanied by lymphocytic alveolitis [82, 83].

Diffuse alveolar haemorrhage presents with three possible histopathologic patterns: pulmonary capillaritis, most frequently encountered in granulomatosis with polyangiitis, bland pulmonary haemorrhage and DAD due to drugs or inhalation of toxic chemicals [84].

Possible diagnostic work-up for this presentation of ILDs is summarised in Fig. 3.

#### Initial evaluation of the severity of respiratory failure and how to choose the site and intensity of care

ARF represents a true clinical and ethical challenge, given the lack of benefit of IMV in the majority of patients with ARF and ILDs [54], the high susceptibility of these patients to ventilator-induced lung injury (VILI) [54, 85], and, subsequently, the high mortality rate of ILD patients in the ICU [86–88]. Despite the majority of studies having been conducted on AE-IPF [86, 87], it appears that AEs of non-IPF forms of ILD do not differ in terms of prognosis [88]; furthermore, the duration of the underlying ILD from diagnosis to ICU admission has not shown to correlate with mortality. The unique definitive treatment option for pharmacologically refractory advanced stage ILDs is lung transplant [89], and the decision to proceed to intensive life support measures in these patients cannot withstand the patient’s precedent enlisting. In rarer cases, a patient may be listed de novo upon ICU admission, in the form of a salvage transplant. To this aim, while waiting for lung transplant, IMV, and more recently ExtraCorporeal Membrane Oxygenation (ECMO) [90], have shown to be lifesaving options. Therefore, patients presenting with ARF on an underlying ILD should be addressed to centers specialized in ILD and disposing of a respiratory high dependency unit (HDU) and an ICU with ECMO experience; in case the patient is on the lung transplant list, he/she should be referred to the local transplant center [89, 91].

In respiratory HDUs, non-invasive ventilation (NIV) is effectively used to decrease work of breathing in patients such as those with exacerbation of chronic obstructive pulmonary disease (COPD). However, in most ILD cases with ARF the excessive work of breathing cannot be managed by NIV alone, and studies suggesting a definitive clinical benefit of NIV in ILDs are still lacking, as shown in the following sections of this review. Furthermore, a recent study on a large single-center cohort of NIV patients, showed that the presence of diffuse ILD was associated with increased mortality [92]. In this scenario, NIV could have a role in selected patients, such as survivors from an AE-ILD, as a bridge to transplant [89, 93], or to reduce patient discomfort after the onset of ARF in those who are not candidate to lung transplant, while establishing other supportive measures [94]. We will discuss specific indications and contraindications together with the available evidence in regards to various oxygenation and ventilation methods in the following sections.

Finally, given the poor prognosis, patients with end-stage ILD with no indication to transplant and aggressive life-support should not die in an acute care setting, but offered palliation and end-of-life supportive care [95]. Furthermore, all patients in advanced stages of ILD that are not candidates to invasive treatments should receive appropriate counseling about the prognosis of the disease and the possible supportive measures available.

In conclusion, site of care in ILD patients with ARF may include a wide range of choices from an ICU with ECMO to palliation centers. Clinicians, with the support of a multidisciplinary team (e.g. pulmunologist and intensivist), should be prepared to promptly recognize a possible candidate to lung transplant from one who may benefit from comfort and supportive measures alone.

### Acute respiratory failure treatment

- Oxygen therapy and high-flow nasal cannula

Oxygen supplementation is the mainstay of treatment of ARF in ILDs. In the acute setting, different kinds of oxygen therapy are available, from simple nasal cannulae to face masks, including high concentration oxygen with reservoir mask and Venturi mask.

Patients with ILDs in advanced stages or during AE have a high degree of diffusion impairment and V/Q mismatch and require high oxygen concentrations to achieve satisfactory respiratory gas exchange. The risk of hypercapnia in these patients is usually minimal, and at the very end stages [96].

In some conditions, patients’ needs can be higher and the oxygen flows provided by conventional therapy may be lower than inspiratory peak flows. Humidified High-Flow Nasal Cannula (HFNC) oxygen therapy is a disposal, which may provide very high flows (up to 60 L/min) and utilizes an air oxygen blend allowing from 21 to 100% FIO2 delivery [97].

This modality of oxygen delivery may offer several advantages in comparison to the usual oxygen therapy, since it provides both steady FIO2, and, theoretically, an anatomical oxygen reservoir within the nasopharynx and oropharynx, by virtue of a carbon dioxide wash-out effect due to high oxygen flow [98–101]. This more efficient carbon dioxide elimination together with reduced dead space results in a reduced ventilatory drive that, ultimately, leads to a decreased respiratory rate and work of breathing [101, 102]. There is also a continuous positive airway pressure (CPAP) effect which provides an upper airway extending pressure that ranges between 3.2 and 7.4 cmH2O with the mouth closed and leads to an increased end-expiratory lung volume [103, 104]. Finally, the heated humidification facilitates secretion clearance, increasing patients’ comfort and maintaining mucosal integrity [105, 106]. Although there is increasing evidence in neonatal and paediatric settings [107], the utility of HFNC in ILDs is unknown.

A recent systematic review analyzed the effect of HFNC vs conventional oxygen therapy or NIV in adults including 11 randomised control trials (RCTs) and concluded that HFNC use was associated with reduction in intubation rate, need for IMV and rate of escalation of respiratory support when compared to conventional oxygen therapy [108]. When HFNC was compared to NIV, no differences in the rate of intubation and escalation of respiratory support was observed. No difference was found in mortality between HFNC and conventional oxygen therapy or NIV utilization. However, inclusion criteria, as well as endpoints and etiology of ARF, were different among studies, leading to the impossibility to draw certain conclusions. In particular, none of the studies explicitly evaluated patients with ILDs.

No studies have yet assessed the effectiveness of HFNC in patients with ARF in ILD, with the exception of a case series. *Horio* et al. described three patients with AE of interstitial pneumonia and unsatisfactory respiratory gas exchange with no rebreathing face masks, who were successfully treated with HFNC [109].

In conclusion, HFNC in ILD patients might potentially be an alternative to conventional oxygen therapy in patients requiring both high concentration of oxygen and high-flow gas to correct hypoxemia and control dyspnea and tachypnea. However, the evidence is very scarce and RCTs are needed to establish the real efficacy of this oxygen delivery method.

b) Non-invasive ventilation

The outcome of patients with ARF and ILDs ventilated in the ICU is very poor and IMV proved to be mostly futile [54]. Current IPF guidelines recommend that the majority of patients with respiratory failure should not receive IMV [46]. The reason of this finding lies in the fact that both the underlying structural lung disease and the precipitating condition causing ARF have a high likelihood of being irreversible and progressive. The pathogenic mechanisms causing AE-ILD have not yet been fully understood, thus, there are no treatments that have proven to be effective in RCTs. In this scenario, NIV may be applied in the hope to treat or stabilize the cause of ARF, minimizing complications and the poor outcome connected with endotracheal intubation (ETI) and IMV [110].

A few studies and case series have evaluated the application of NIV in ARF in ILDs. Two case series by *Yokoyama and colleagues* and *Vianello* et al. reported similar results in IPF patients with ARF [111, 112]. On one hand, a minority of patients benefited from NIV application (5/11–45% - and 8/18–44%, in the cohorts of *Yokoyama* and *Vianello*, respectively), and, in those who responded, the use of NIV was associated with better outcomes, including avoidance of ETI and better survival. On the other hand, patients who failed NIV died within 3 months, regardless of ETI.

*Gungor and colleagues* evaluated the use of NIV both in IPF and in other ILDs with better overall prognosis, including CTD and silicosis [113], however, they found similar outcomes to the previously cited cohorts of IPF patients [111, 112], with a higher mortality rate and NIV failure in those who needed continuous NIV compared to those that were able to tolerate NIV interruptions for feeding.

NIV responsiveness was associated with less severe ARF and patients’ condition in most series [94, 111–114]. Furthermore, *Yokoyama* et al. in a study on 38 patients with ARF in ILDs, including IPF, CTD-ILD and drug-induced ILD, reported that early NIV initiation during the acute phase was associated with better 30-day survival [114], indicating that early optimization of supportive care, including oxygenation, may improve patients’ management and short-term outcomes.

A further step was attempted by *Aliberti and colleagues* who evaluated NIV responsiveness in patients with ILD and ARF in a multicentre study, stratifying the results according to the cause of ARF and the underlying radiological pattern of ILD [115]. NIV showed to improve oxygenation in patients with pneumonia, but not in those with AE; however, this positive effect on oxygenation did not translate into improved outcomes in patients with pneumonia compared to those with AE. No differences were observed in terms of radiological pattern. These results seem to suggest that, in spite of a better oxygenation in specific subgroups of patients, NIV outcomes do not depend on the radiological pattern or underlying cause of ARF. In fact, even if the precipitating factors are promptly recognized and treated, most cases show an irreversible deterioration of baseline conditions.

In conclusion, despite the high rate of NIV failure in patients with ARF in ILDs, in selected patients, such as those with less severe respiratory failure, an early NIV trial might facilitate the recognition of NIV-responder patients, who may present better short-term clinical outcomes. However, NIV responsiveness does not seem to impact on the poor prognosis related to the underlying disease: one-year mortality rate in NIV-responder patients was ≥70% in all the studies evaluated [94, 111, 112].

The main difficulties encountered when applying NIV on patients with ILDs and some practical tips to overcome them are summarised in Table 2.

c) Invasive mechanical ventilation

**Table 2:** Clinical aspects of Non-Invasive Ventilation in Interstitial Lung Diseases

Problem	Tip for solution
High pressures required to obtain ideal tidal volume in fibrotic lung with risk of pneumothorax	- Tolerate low tidal volumes with higher respiratory rate to obtain acceptable minute ventilation - Low to moderate PEEP levels to avoid overdistension of “healthy” lung units
High respiratory rate that hampers patient-ventilator adaptation	- Titrate drugs to control respiratory rate, e.g. opiates (morphine or fentanyl)^§^
Intense breathlessness reported by patients especially in the acute phase	- Titrate drugs to control respiratory rate, e.g. opiates (morphine or fentanyl)^§^ - Rapid inspiratory curve - Increase FIO2

Footnotes: PEEP = positive end expiratory pressure; FIO2 = fraction of inspired oxygen

§ Matsumoto T, Tomii K, Tachikawa R, Otsuka K, Nagata K, Otsuka K, et al. Role of sedation for agitated patients undergoing noninvasive ventilation: clinical practice in a tertiary referral hospital. Bmc Pulm Med. 2015 Jul 13;15:71

During an episode of severe ARF, patients might need intensive care support, including ETI and IMV. Multiple retrospective studies have taken into account patients with IPF admitted to the ICU for ARF [11, 54, 55, 86, 87, 116, 117]. In all the reported studies, IMV was associated with negative clinical outcomes, in some cases with an in-hospital mortality higher than 90%. In some cohorts causes of ARF were divided into reversible, such as pneumonia, and irreversible, mainly AE-ILD. In the absence of reversible causes, patients did not seem to benefit from IMV, and, in these cases, some authors advised against the use of IMV, with the exception of patients listed for lung transplant [55].

A study by *Mollica* et al. in 2010 compared IMV to NIV or spontaneous breathing in patients with ARF and IPF [94]. Although both ventilatory strategies improved PaO2/FIO2 ratio, in comparison to spontaneous breathing, the authors reported a global in-hospital mortality of 85%, that rose up to 100% in patients treated with IMV.

Other authors evaluated the use of IMV in ARF in non-IPF fibrotic ILDs. *Molina-Molina* et al. assessed 20 patients with fibrotic ILDs (14 with IPF and 6 with CTD-ILD) who required IMV [118]: similarly to cohorts with only IPF patients, IMV was associated with 100% in-hospital mortality.

*Vincent* et al. and *Gaudry and colleagues*, described the same scenario from a different point of view [119, 120]. Both authors described IPF and fibrotic-NSIP patients with ARF treated with NIV or IMV. *Vincent* et al. observed that by dividing their cases into two successive time periods (the first period from 1999 to 2004 and the second from 2005 to 2009), the patient’s prognosis improved in the second time period compared to the first [119]. Despite all the limitations of this study, the authors hypothesized that some of the improvements introduced in the years, including lung-protective ventilatory parameters with lower tidal volumes and plateau pressures and earlier admission to the ICU, might have changed the outcomes of these patients, suggesting that not all patients are doomed to poor short-term outcomes. *Gaudry* et al., while observing a poor prognosis in most cases (21 out of 27 patients, 85%, died during hospitalization), found that a small subgroup of patients survived (6 cases) and some of them were able to reach pulmonary transplant (2 patients, 7%), confirming that IMV might be indicated in very selected patients candidate to lung transplant [120].

One of the mechanisms involved in the poor prognosis of mechanically ventilated patients with advanced fibrotic ILDs is VILI [88, 120]. The forces applied through IMV can cause pathophysiological alterations culminating in the disruption of cell membranes and cell-cell contacts, particularly in those patients with inhomogeneously injured lungs, such as in ARDS [121]. The fibrotic lung might be subject to a similar VILI as a consequence of baro- and volutrauma. *Nava and colleagues* assessed the respiratory mechanics of end-stage fibrotic ILDs during IMV and reported an approximately fourfold increase in the elastance of the respiratory system compared to normal anaesthetised subjects [122]; the values reported were even higher than those in patients with ARDS [122]. This marked increase in lung elastance is mainly due to the “stiffness” of the fibrotic lung. At the same time, the authors also observed that the resistance of the respiratory system was markedly increased compared with the values reported for normal subjects (although to a lesser extent than COPD patients) [122]. This finding reflects similar observations by *West and colleagues* that reported in patients with fibrotic lung diseases an increased resistive work in association with a higher work of breathing in comparison to normal subjects [123].

In conclusion, despite the low quality of the evidence available, IMV in patients with IPF and advanced fibrotic ILDs that develop ARF seems to be contraindicated, because of the high short-term mortality. However, it is necessary to differentiate some subgroups of patients, such as those with a potentially reversible cause of ARF or those listed for lung transplant, on whom prognostic data are less accurate and indications to IMV should be assessed on a case-by-case basis.

d) Extracorporeal membrane oxygenation

ECMO, referring to an extracorporeal circuit that directly oxygenates and removes carbon dioxide from the blood, may be considered in refractory severe ARF when positive-pressure ventilation, in combination with other ventilatory strategies such as prone positioning and neuromuscular block, results in unacceptable levels of hypoxemia, hypercapnia and acidemia [124].

ECMO in ILDs might be considered in patients with severe respiratory failure secondary to a potentially reversible cause of deterioration (e.g., infection or pulmonary embolism) and if the patient is a candidate for lung transplant [125].

The recent improvements in ECMO technology have allowed some centres to use less invasive ECMO or other extracorporeal devices to liberate patients from IMV and successfully bridge them to lung transplantation [125].

*Trudzinski* et al. reported their experience with ILD patients treated with ECMO for ARF [90]. ECMO was only used when patients were considered potential candidates for lung transplant or when two intensivists agreed on a potentially reversible pulmonary cause of ARF (e.g., acute infection on previous chronic ILD). Indications for veno-venous ECMO in the 21 patients included in the study were: refractory hypoxemia or uncompensated hypercapnia in ARDS despite optimization of IMV, or refractory hypoxemia despite maximal non-invasive therapies in patients considered “at risk of intubation”. In the latter case, ECMO was used to prevent intubation, and referred to as “awake-ECMO”, meaning the use of ECMO in awake, non-intubated, spontaneously breathing patients. The authors reported that out of the 21 patients treated with ECMO, six (29%) underwent lung transplant and two (10%) died on the waiting list after 9 and 63 days on ECMO. Of the 15 patients who did not undergo lung transplantation, 14 died after a mean time on ECMO of 40 days. These results confirm that in the absence of effective therapeutic perspectives (e.g. transplantation), ECMO is unable to prevent the irreversible progression of the underlying disease and does not improve mortality in patients with severe ARF and ILDs.

*Fuehner* et al. were the first to compare the outcomes of awake-ECMO patients with terminal respiratory or cardiopulmonary failure candidate to lung transplant to a historical cohort of patients treated with conventional IMV as bridge to transplant [126]. The duration of awake-ECMO and IMV were similar between the 2 groups (median 9 and 15 days, respectively), as well as the mortality rate on ECMO and IMV before a donor organ was available (23 and 29%, respectively). Results were encouraging, with a 6-month survival rate after transplantation of 80% in the “awake-ECMO” group vs 50% in the mechanically ventilated group.

A strategy based on ECMO to avoid IMV might potentially offer numerous benefits, including prevention of VILI and ventilator-associated pneumonia, preservation of oral feeding, spontaneous coughing, and social interaction, and allowance of early rehabilitation [127]. However, extracorporeal support devices are frequently linked to complications, including vessel perforation, bleeding, and infections. The main complications reported in *Trudzinski*’s cohort were bleeding (3 cases, 14%) and cannulation placement (2 cases, 13%), while in *Fuehner*’s cohort were bleeding requiring transfusions (8 cases, 31%), sepsis-like syndrome (5 cases, 19%), intractable coughing requiring ETI (2 cases, 8%).

Of all the available forms of extracorporeal gas exchange, partial lung support, also known as extracorporeal CO_2_ removal (ECCO2R) or respiratory dialysis, has shown some interesting results [128]. Recently, ECCO_2_R has been proposed as an intervention to eliminate CO2 from the blood of patients undergoing NIV who are unable to achieve adequate gas exchange despite optimization of ventilatory settings [129]. *Vianello* et al. described the successful management of an IPF patient with ARF using a pump-assisted veno-venous system for ECCO_2_R as an alternative to ETI following NIV failure [130]. The authors were able to minimize the complications most commonly related to ECMO by using a small single veno-venous dual lumen catheter. However, both clinicians’ experience and the technology behind ECCO_2_R still need to be improved before its use can be widely implemented in clinical practice.

In conclusion, extracorporeal lung support might allow to prevent or reduce the invasiveness of IMV, and, therefore, minimize the risk of “triggering” fatal deterioration of the underlying chronic process, such as VILI. However, ECMO alone does not change the poor outcome associated with severe ARF in ILDs, and, given the high costs and risk of complications, should be limited to patients with a potential good short-term prognosis, e.g. those listed for lung transplant.

## Conclusions

ARF is a feared complication in ILDs, both for its difficult management and diagnostic work-up and the poor prognosis.

Oxygen supplementation and ventilatory support have proven to be ineffective in modifying the prognosis of the disease in the absence of effective therapeutic options. Less invasive techniques, including HFNC oxygen and NIV, might be used in less severe cases to correct hypoxemia and control dyspnea, while, invasive techniques, such as IMV and ECMO, should be limited to patients listed for lung transplant or with reversible causes of ARF.

## Abbreviations

AE: Acute exacerbation
AE-IPF: AE of IPF
AEP: Acute eosinophilic pneumonia
AFOP: Acute fibrinous organizing pneumonia
AIP: Acute interstitial pneumonia
ARDS: Acute respiratory distress syndrome
ARF: Acute respiratory failure
BAL: Bronchoalveolar lavage
CHP: Chronic hypersensitivity pneumonitis
COP: Cryptogenic organizing pneumonia
COPD: Chronic obstructive pulmonary disease
CPAP: Continuous positive airway pressure
CTD-ILD: Connective tissue disease related ILD
DAD: Diffuse alveolar damage
DAH: Diffuse alveolar hemorrhage
ECCO2R: Extracorporeal CO_2_ removal
ECMO: ExtraCorporeal Membrane Oxygenation
ETI: Endotracheal intubation
HDU: High dependency unit
HFNC: High-Flow Nasal Cannula
HRCT: High-resolution computed tomography
ICU: Intensive care unit
ILD: Interstitial lung disease
IMV: Invasive mechanical ventilation
IPF: Idiopathic pulmonary fibrosis
NIV: Non-invasive ventilation
NSIP: Non-specific interstitial pneumonia
RCT: Randomised control trial
V/Q: Ventilation-perfusion
VILI: Ventilator-induced lung injury
